# Activation of Src Mediates PDGF-Induced Smad1 Phosphorylation and Contributes to the Progression of Glomerulosclerosis in Glomerulonephritis

**DOI:** 10.1371/journal.pone.0017929

**Published:** 2011-03-22

**Authors:** Akira Mima, Hideharu Abe, Kojiro Nagai, Hidenori Arai, Takeshi Matsubara, Makoto Araki, Kazuo Torikoshi, Tatsuya Tominaga, Noriyuki Iehara, Atsushi Fukatsu, Toru Kita, Toshio Doi

**Affiliations:** 1 Department of Nephrology, Institute of Health Biosciences, University of Tokushima Graduate School, Tokushima, Japan; 2 Department of Geriatric Medicine, Kyoto University Graduate School of Medicine, Kyoto, Japan; 3 Department of Nephrology, Kyoto University Graduate School of Medicine, Kyoto, Japan; 4 Department of Cardiovascular Medicine, Kyoto University Graduate School of Medicine, Kyoto, Japan; Ohio State University, United States of America

## Abstract

Platelet-derived growth factor (PDGF) plays critical roles in mesangial cell (MC) proliferation in mesangial proliferative glomerulonephritis. We showed previously that Smad1 contributes to PDGF-dependent proliferation of MCs, but the mechanism by which Smad1 is activated by PDGF is not precisely known. Here we examined the role of c-Src tyrosine kinase in the proliferative change of MCs. Experimental mesangial proliferative glomerulonephritis (Thy1 GN) was induced by a single intravenous injection of anti-rat Thy-1.1 monoclonal antibody. In Thy1 GN, MC proliferation and type IV collagen (Col4) expression peaked on day 6. Immunohistochemical staining for the expression of phospho-Src (pSrc), phospho-Smad1 (pSmad1), Col4, and smooth muscle α-actin (SMA) revealed that the activation of c-Src and Smad1 signals in glomeruli peaked on day 6, consistent with the peak of mesangial proliferation. When treated with PP2, a Src inhibitor, both mesangial proliferation and sclerosis were significantly reduced. PP2 administration also significantly reduced pSmad1, Col4, and SMA expression. PDGF induced Col4 synthesis in association with increased expression of pSrc and pSmad1 in cultured MCs. In addition, PP2 reduced Col4 synthesis along with decreased pSrc and pSmad1 protein expression in vitro. Moreover, the addition of siRNA against c-Src significantly reduced the phosphorylation of Smad1 and the overproduction of Col4. These results provide new evidence that the activation of Src/Smad1 signaling pathway plays a key role in the development of glomerulosclerosis in experimental glomerulonephritis.

## Introduction

Glomerulonephritis is usually progressive and remains an important cause of end stage renal disease. In sclerosing glomerulonephritis, accumulation of the extracellular matrix (ECM) is a critical process in progressive glomerular injuries [1], [2]. Type IV collagen (Col4) is one of the most important components of the expanded ECM [3]. Moreover, smooth muscle α actin (SMA) is a known common molecular marker of phenotypic changes of mesangial cells (MCs) in many glomerular diseases. We previously reported that Smad1 participates in the development of glomerulosclerosis in experimental glomerulonephritis [4]. We also reported that Smad1 transcriptionally regulates the expression of Col4 and SMA [5], [6]. However, the mechanisms by which Smad1 is activated in glomerulonephritis have not been fully elucidated.

Platelet-derived growth factor (PDGF) is known to be a critical mitogen for MCs in vitro and in vivo [1], [7]. It is noteworthy that mice deficient for PDGF B or PDGF receptor show abnormal glomeruli due to a lack of MC development [8]–[11]. Several lines of evidence indicate that PDGF plays a key role in the development of glomerulosclerosis not only in experimental models but also in human glomerular diseases [12], [13]. The introduction of a neutralizing anti-PDGF antibody has shown that both mesangial proliferation and glomerulosclerosis can be markedly ameliorated in a rat glomerulonephritis model [14]. Moreover, we previously showed that the development of glomerulosclerosis from mesangial proliferation is dependent on PDGF-induced Smad1 activation [4], but little is known concerning the regulatory mechanisms of Smad1 activation by PDGF in glomerulonephritis. c-Src is a ubiquitously expressed non-receptor protein-tyrosine kinase [15] that is involved in multiple pathways regulating cell growth, migration, and survival [16]. c-Src is also an important component of the PDGF signal transduction pathway [17]. Several reports have demonstrated that PDGF plays a key role in MC proliferation and glomerulopathy *in vivo* and *in vitro* [7], [18], [19]. Previously we demonstrated that Smad1 is phosphorylated by PDGF in MCs [4]. However, the exact role of c-Src in MCs as well as in glomerulonephritis remains unclear.

In the present study, we demonstrated that c-Src is activated in experimental proliferative glomerulonephritis and that the reduction of c-Src ameliorates the development of glomerulosclerosis by blocking of the Smad1 signal transduction pathway. We further showed that c-Src plays an important role as a switch molecule for the activation of Smad1 downstream of PDGF signaling. These findings unveil the molecular mechanisms underlying the induction of MC proliferation and MC phenotype alteration, resulting in proliferative glomerulonephritis. Taking these results together, we hypothesized that the Src/Smad1 pathway may be critical in the pathogenesis of proliferative glomerulonephritis.

## Materials and Methods

### Animals

Full details of the animal experimental protocols were approved and ethical permission was granted by the Review Board of Kyoto University (Permit Number: Med Kyo 08508). We used age-matched male Wistar rats (8 to 12 weeks old, 180 to 200 g) bred at the Shimizu Laboratory Animal Center (Hamamatsu, Japan). The animals were housed under specific pathogen-free conditions at the Animal Facility of Kyoto University. Levels of serum creatinine and blood urea nitrogen were measured using a Hitachi Mode 736 autoanalyzer. The urinary albumin concentrations were measured from 24-h urine collections by Nephrat and Albuwell (Exocell), according to the manufacturer's protocols.

### Cell culture experiments

A glomerular mesangial cell line was established from glomeruli isolated from normal 4-week-old mice (C57BL/6JxSJL/J) and was identified according to a method described previously [7]. The MCs were plated on 100-mm plastic dishes (Nunc) that were maintained in B medium (a 3∶1 mixture of minimal essential medium/F12 modified with trace elements) supplemented with 1 mM glutamine, penicillin at 100 units/ml, streptomycin at 100 µg/ml, and 10% fetal calf serum (Irvine Scientific). The cells were passaged weekly with trypsin-EDTA. The cultured cells fulfilled the previously described criteria generally accepted for glomerular mesangial cells [20]. Stimulation with angiotensin II (Ang II) (Sigma), PDGF, PP2 (Calbiochem, Darmstadt, Germany), or olmesartan (Cosmo Bio, Tokyo, Japan) was carried out in DMEM containing 0.5% FCS at 37°C for the indicated times. A rat monoclonal anti-PDGFβ-receptor antibody (APB5) and its antagonistic effects on the PDGFβ-R signal transduction pathway in vitro have been described previously [4].

### Constructs, transfection, and co-immunoprecipitation

Src cDNAs (pUSE Src wild type, pUSE Src kinase mutant, and empty vector) were obtained from Upstate Biotechnology, Inc. (Lake Placid, NY). MCs were transfected using FuGene6 (Roche, Mannheim, Germany) according to the manufacturer's protocol. After 48 h of transfection, the cells were washed with PBS, and 1 ml ice-cold lysis buffer (25 mM Tris-HCl pH 7.4, 100 mM NaCl, 2 mM EDTA, 0.5% Nonidet P-40, Complete protease inhibitors cocktail; Roche) was added. For co-immunoprecipitation assay, whole cell lysates were first pre-cleared with protein G–Sepharose (Amersham) and followed by incubation with anti-PDGFR antibody (Santa Cruz) for 3 h at 4°C. The immune complex was isolated and separated by SDS–PAGE and analyzed by Western bot analysis. Protein was detected using polyclonal rabbit anti-Src antibody (Cell Signaling Technology).

### Histology and Immunohistochemistry

Tissues were fixed in Methyl Carnoy's solution and were paraffin-embedded. Multiple sections were prepared and stained with periodic acid silver methenamine (PASM) and periodic acid-Schiff's reagent (PAS). Immunohistochemical staining was performed with antibodies specific to Col4 (Progen) or SMA (Abcam), using an established avidin-biotin detection method (Vector Laboratories). Frozen sections were used for the detection of pSrc and pSmad1 (Cell Signaling Technology). Glomerular morphometry was evaluated in PASM-stained tissues. The glomerular surface area and the PASM-positive area/glomerular area (%) were measured using an image analyzer with a microscope (IPAP, Sumitomo Chemical, Osaka, Japan) as previously described [21]–[24]. To quantitatively measure the expression of pSrc and pSmad1, pSrc-positive or pSmad1-positive cells/DAPI-positive nuclei were counted, and the mean percentages of pSrc-positive or pSmad1-positive cells were calculated. An investigator scored sections in a blinded fashion, according to an established scoring system (range 0–4; 0, no ECM deposition; 4, ECM deposition in all sections of the glomeruli) to semiquantify the localization of Col4 and SMA.

### Small-interfering RNA

MCs (0.5×10^5^) were seeded into 12-well plates (Nunc) and were grown until they were 60% to 80% confluent. The small-interfering RNAs (siRNAs) for c-Src, Smad1, and LRP1 (Dharmacon) or control scrambled siRNA (Dharmacon) were combined with DharmaFECT transfection reagent (Dharmacon), and the cells were transfected according to the recommended protocol with siRNA (100 nM final concentration). After 48 h of transfection, cells were starved in DMEM containing 0.5% BSA before treatment. After 48 h of incubation, the cells were stimulated with or without PDGF (Calbiochem).

### TGFβ-neutralizing antibody assay

MCs were resuspended at a concentration of 1×10^6^ cells/ml and plated onto 100-mm dish either in the presence of 10 µg/ml TGFβ-neutralizing antibody (R&D Systems) or a control normal chicken IgY. After 24 h of incubation, the cells were treated with PDGF for additional 12 h and were harvested and underwent protein extraction on Western blotting.

### Western blotting

Isolated glomerular MCs were suspended in RIPA buffer (50 mM Tris, pH 7.5, 150 mM NaCl, 1% Nonidet P-40, 0.25% SDS, 1 mM Na_3_VO_4_, 2 mM EDTA, 1 mM phenylmethylsulfonyl fluoride, 10 mg/ml of aprotinin) and incubated for 1 h at 4°C. After centrifugation, the supernatants were used as total cell lysates. Twenty micrograms of each sample was applied to SDS-PAGE. After electrophoresis, the proteins were transferred to nitrocellulose filters (Schleicher & Schuell). The blots were subsequently incubated with anti-phospho-Smad1, anti-phspho-Src (Cell Signaling Technology), anti-SMA, anti-LRP1 (Abcam) or anti-Col4 antibody (Progen), followed by incubation with horseradish peroxidase-conjugated goat anti-rabbit IgG and sheep anti-mouse IgG (Amersham). The immunoreactive bands were visualized using horseradish peroxidase-conjugated secondary antibody and the enhanced chemiluminescent system (Amersham). These bands were quantified using an imaging densitometer (Science Lab 99 Image Gauge, Fujifilm, Tokyo, Japan).

### Data analysis

The data are expressed as the mean ± S.D. Comparison among more than two groups was performed by one-way analysis of variance (ANOVA), followed by post hoc analysis (Bonferroni/Dunn test) to evaluate the statistical significance between the two groups. All analyses were performed using StatView (SAS Institute, Cary, NC). Statistical significance was defined as *P*<0.05.

## Results

### Glomerular phosphorylation of c-Src and Smad1 parallels the progress of glomerulosclerosis in rat Thy1 GN

**Figure 1:**
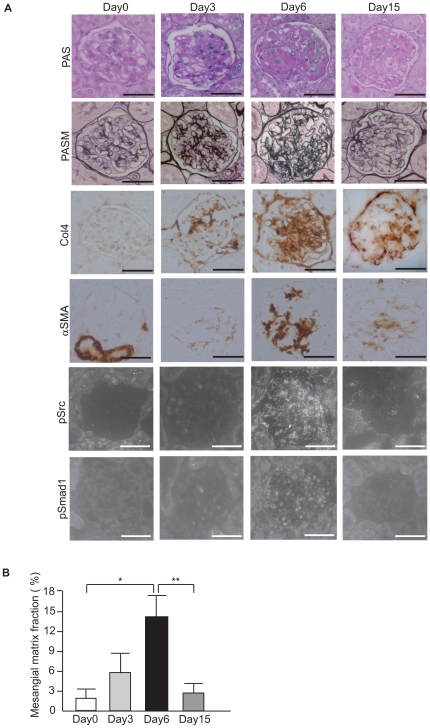
Induction and activation of c-Src and Smad1 in proliferative glomerulonephritis. (A) Representative light-microscopic appearance and immunohistochemistry of glomeruli in Thy1 GN. Scale bars = 100 µm. (B) Quantitative assessment of PASM staining in Thy1 GN. *P = 0.002, **P = 0.002).

**Figure 2:**
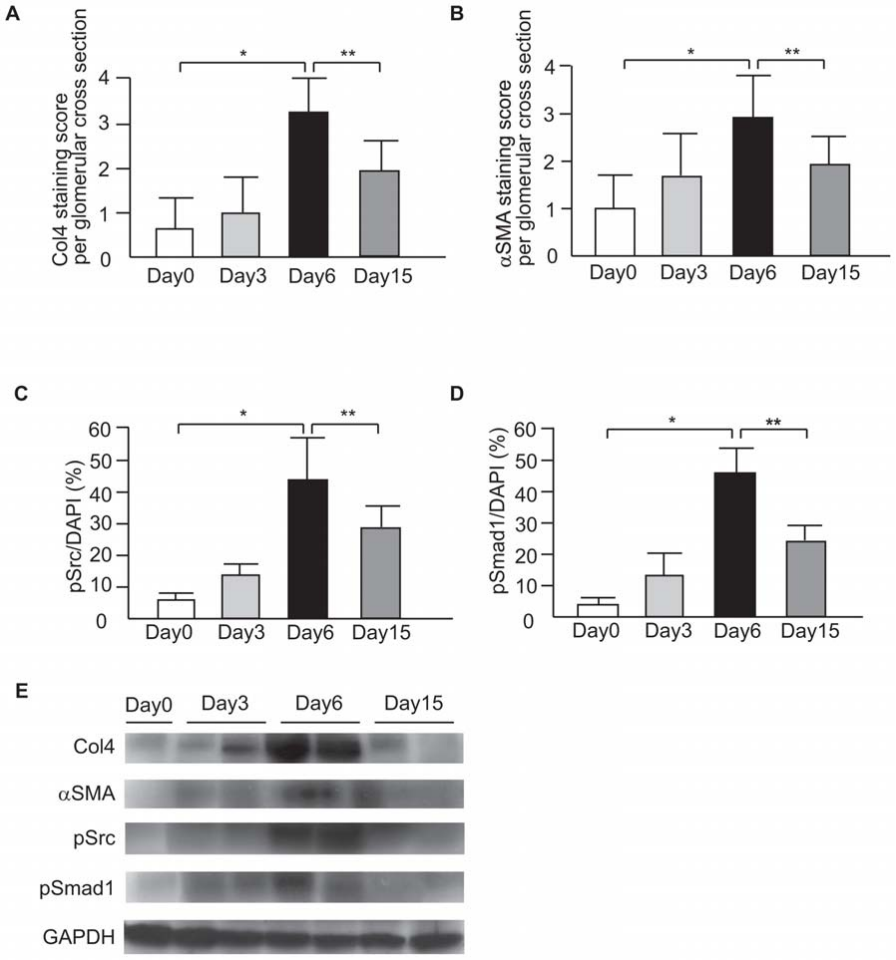
Time course of glomerular expression of Col4, SMA, pSrc and pSmad1 in Thy1 GN. (A, B) Staining scores per glomerular cross-section for Col4 (*P<0.001, **P<0.001) and SMA (*P<0.001 and **P = 0.009) were calculated. Data represent mean values ± S.D. of at least three independent experiments; n = 6 for each experimental group. (C, D) Quantification of glomerular pSrc and pSmad1 by optical densitometry. The pSrc-positive nuclei and pSmad1-positive nuclei were counted in 10 consecutive fields in each specimen and normalized by the number of DAPI-positive nuclei. *P<0.001, **P<0.001. (E) Western blot for the glomerular lysates from each group. Data represent mean values ± S.D. of at least three independent experiments; n = 6 for each experimental group.

We utilized a model of mesangial proliferative glomerulonephritis, known as anti-Thy1-induced glomerulonephritis (Thy1 GN), which exhibits sclerosis in the glomeruli. The renal function of Thy1 GN on day 6 was significantly decreased (Figure S1A). MC proliferation began on day 3 and glomerulosclerosis began on day 6. Renal damage clearly regressed until day 15. Sclerosis in the kidney peaked on day 6 and sclerotic changes subsided until day 15 (Figure 1A and B). Localization of phospho-Src (pSrc) and phospho-Smad1 (pSmad1) in the nuclei was scant on day 0. On day 3, phosphorylation began in c-Src and Smad1 proteins. The level of phosphorylation gradually increased and positively stained nuclei in parallel with the activity of mesangial proliferation during the development of glomerulosclerosis. Phosphorylation peaked on day 6 and then decreased towards day 15 (Figure 2, C, D and E). Phosphorylation of c-Src and Smad1 was almost undetectable on day 0 but became prominent during the proliferative stages in Thy1 GN, peaked on day 6, and then decreased towards day 15 (Figure 2C, D and E). In addition, the expression of Col4 and SMA changed in parallel with the activation of c-Src and Smad1 (Figure 2A, B and E). These data suggest that both Smad1 and c-Src are activated in the course of proliferative injuries in rat kidneys.

### PP2 preserves renal function and attenuates glomerulosclerosis in rat glomerulonephritis

**Figure 3:**
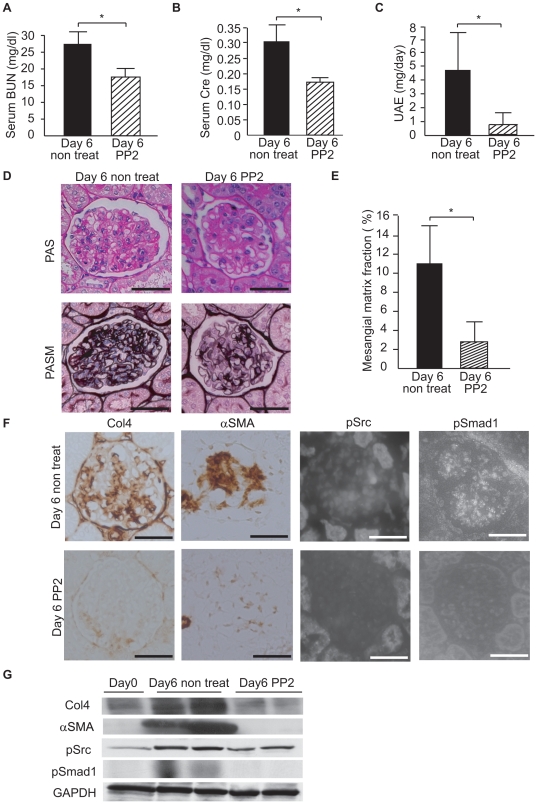
Src-specific inhibitor PP2 inhibits glomerulosclerosis and glomerular expression of pSrc and pSmad1 in Thy1 GN. (A–C) Serum blood urine nitrogen (BUN), serum creatinine (Cre), and UAE in the nontreatment and PP2 groups. *P* values were 0.001, 0.001 and 0.017, respectively. (D, E) Representative light-microscopic appearance of glomeruli (PAS and PASM staining) and quantitative assessment of PASM staining in Thy1 GN with or without PP2 on day 6. Scale bars = 100 µm. *P<0.001. (F) Immunohistochemistry of glomeruli (Col4, SMA, pSrc and pSmad1) in Thy1 GN with or without PP2 on day 6. Scale bars = 100 µm; n = 6 for each experimental group. (G) Western blot for the glomerular lysates from each group. Data represent mean values ± S.D. of at least three independent experiments; n = 6 for each experimental group on day 6.

To investigate whether the c-Src/Smad1 pathway plays a pivotal role in developing glomerulosclerosis, we administered a Src specific inhibitor, PP2, to Thy1 GN rats from days 0 to 6 and assessed glomerulosclerosis on day 6. Untreated Thy1 GN rats showed an increased degree of glomerulosclerosis, whereas glomerulosclerosis was significantly decreased in the PP2-treated group (Figure 3A, B), along with renal function (Figure 3, C–E).

### PP2 represses the activation of Smad1 and the expression of both Col4 and SMA in rat glomerulonephritis

Next, to examine the effect of PP2 on the morphological changes seen in Thy1 GN glomerulosclerosis, we examined Col4 and SMA expression in the two groups. PP2 treatment significantly inhibited Col4 and SMA expression, whereas expression was increased in the non-treatment group (Figure 3F). Moreover, we examined whether PP2 affected the phosphorylation and translocation of c-Src and Smad1 in Thy1 GN rats. PP2 treatment inhibited the phosphorylation of c-Src and Smad1, and their expression was localized in the nucleus in untreated Thy1 GN (Figure 3F). These data from immunohistochemistry were confirmed by Western blot analysis (Figure 3G).

### Effect of PP2 on PDGF-mediated signaling in MCs

**Figure 4:**
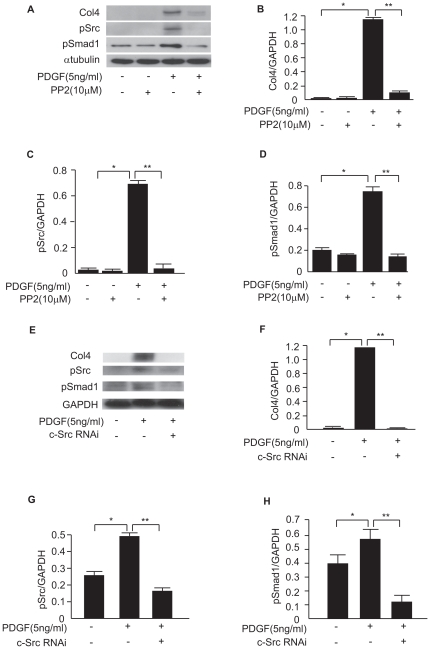
Activation of c-Src and Smad1 is regulated by PDGF in MCs. (A) Effect of PP2 on pSrc, pSmad1 and Col4. MCs were preincubated with PP2 (10 µM) or DMSO for 48 h before exposure to PDGF (5 ng/ml, 12 h). (B) Optical densitometry of Col4 in western blot. *P<0.001 and **P<0.001. (C, D) Optical densitometry of pSrc (*P<0.001 and **P = 0.003) and pSmad1 (*P = 0.002, **P = 0.002) in western blot analyses. (E) Effects of RNAi-mediated silencing of c-Src on pSrc, pSmad1 and Col4 under stimulation of PDGF (5 ng/ml, 12 h). (F–H) Optical densitometry of Col4 (*P<0.001, **P<0.001), pSrc (*P<0.001, **P<0.001), and pSmad1 (*P = 0.02, **P = 0.002) in western blot. Data represent mean values ± S.D. of at least three independent experiments.

Because PDGF is well known to play a key role in the development of glomerulosclerosis, we investigated whether PDGF can activate c-Src/Smad1 signal transduction and increase the synthesis of Col4. Expression of Col4, pSrc, and pSmad1 was induced by PDGF stimulation in MCs cultured for 12 hours (Figure 4A–D). These inductions were inhibited by PP2 treatment (Figure 4A–D). These results indicate that PDGF induced the expression of Col4 through the activation of Src/Smad1 signal transduction.

### Silencing of c-Src in MCs inhibits PDGF-mediated phosphorylation of Smad1 and synthesis of Col4

To further confirm the role of c-Src in PDGF-induced upregulation of Smad1 and Col4 expression, c-Src gene silencing by siRNA was performed. c-Src silencing suppressed the PDGF-induced phosphorylation of Smad1 and the synthesis of Col4. In contrast, GAPDH protein levels, used as a loading control, were not affected across the samples (Figure 4E–H). We confirmed the result of knockdown experiments with PDGF stimulation by using three c-Src siRNAs (Src siRNA-1, -2, and -3) (Figure S2). We showed the representative data from using Src siRNA-3 in Figure 4E–H. From these results, c-Src may be significantly involved in PDGF-mediated Col4 expression.

### Activated c-Src is associated with PDGFR in MCs

**Figure 5:**
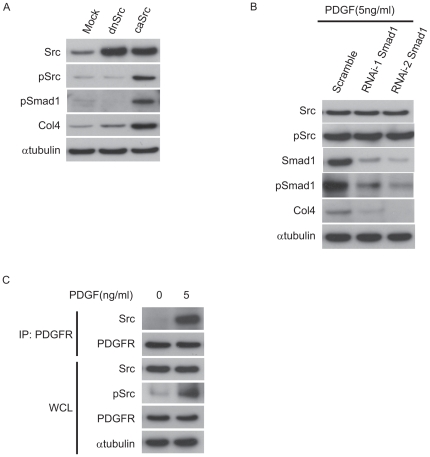
Activated c-Src is associated with PDGF Receptor (PDGFR) in MCs. (A) Western blot analyses of MCs transfected with constitutively active c-Src (caSrc), dominant negative c-Src (dnSrc), and empty vector (Mock). One of three independent experiments is shown. (B) Effects of RNAi-mediated silencing of Smad1 on pSmad1 and Col4 after 5 h stimulation of PDGF (5 ng/ml). Scrambled siRNA (Scramble) was used as a control. One of three independent experiments is shown. (C) MCs were serum-starved for 10 h and then incubated with 5 ng/ml of PDGF for 5 min. Whole cell lysates (WCL) were immunoprecipitated with polyclonal anti-PDGFR antibody and subjected to anti-Src immunoblot.

To clarify the intracellular interaction between PDGF signaling pathway and c-Src/Smad1 axis, the effects of constitutively active form of c-Src (caSrc) transfected in MCs was examined. Transient transfection of MCs with caSrc could induce phosphorylation of Smad1 wihtout stimulation of PDGF, and subsequently upregulated Col4 expression (Figure 5A). In contrast, transfection of the dominant negative Src (dnSrc) did not show these regulations. Moreover, we performed knockdown analysis using Smad1 siRNAs to confirm the role of Smad1 in the regulatory effect of PDGF-induced Col4 expression. Knockdown study revealed that Smad1 acts downstream of PDGF-c-Src signaling pathway in the induction of Col4 (Figure 5B). Furthermore we have explored the possibility that c-Src, while interacting directly with PDGF receptor, could transduce the PDGF signals in MCs. For this purpose, PDGF receptor was immunoprecipitated from whole cell lysates after PDGF stimulation. Anti-c-Src immunoblot revealed that c-Src really associates with PDGFR only when stimulated by PDGF (Figure 5C).

### TGFβ signaling pathway partially mediated PDGF-induced Smad1/Col4 expression in MCs

**Figure 6:**
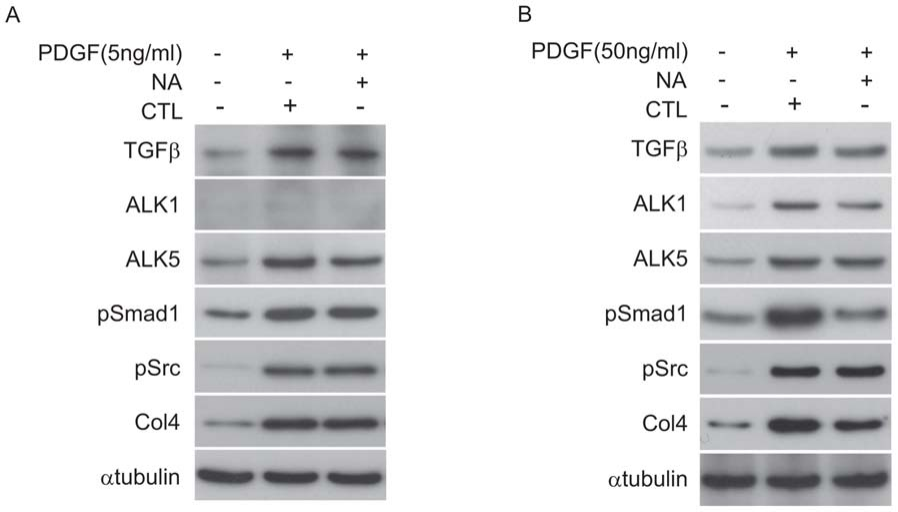
PDGF modulated TGFβ-Activin Receptor-like Kinases (ALKs) signaling pathways in MCs. (A, B) MCs were treated with neutralizing antibody for TGFβ (10 µg/ml) (NA) or control normal IgY (CTL) for 24 h prior to treatment with PDGF at indicated concentrations for 24 h. Equal amounts of cell lysates were subjected to Western blot. One of three independent experiments is shown.

Transforming growth factor beta (TGFβ) is an important growth factor in the modulation of cell proliferation as well as PDGF in a variety of cells. In addition, several studies reported that PDGF may increase the production of TGFβ and the expression of TGFβ type I receptor [25], [26]. To elucidate the the molecular basis of the influence of PDGF on TGFβ signaling pathway, we performed TGFβ-neutralizing antibody assay for PDGF-stimulated MCs. PDGF increased the expressions of TGFβ and activin receptor-like kinase 5 (ALK5) and activated Smad1. However, these changes by PDGF could not be inhibited by neutralizing anti-TGFβ antibody (Figure 6A), indicating that PDGF, but not TGFβ, upregulates expression of ALK5, pSmad1, pSrc, and Col4. In particular, pSmad1 is phosphorylated by ALK1, but not by ALK5, therefore, we investigated the effects of high concentration of PDGF on MCs. At concentration of 50 ng/ml, PDGF increased the expressions of ALK1 as well as other proteins (Figure 6B). Interestingly, an addition of neutralizing anti-TGFβ antibody suppressed not only ALK1 expression, but also expressions of pSmad1 and Col4 (Figure 6B). These results suggest that PDGF has the potential to enhance TGFβ signal transduction through ALK1 as well as ALK5.

### TGFβ signaling pathway partially mediated PDGF-induced Smad1/Col4 expression in MCs

**Figure 7:**
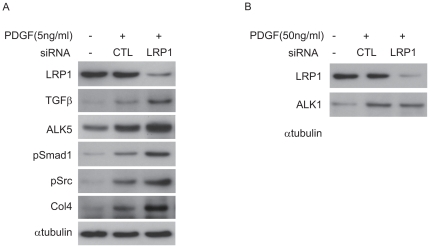
LRP1 modulated both PDGF and TGFβ signaling pathways in MCs. (A, B) Effects of PDGF stimulation and RNAi-mediated silencing of LRP1 after 5 h stimulation of PDGF at indicated concentrations on MCs. Scrambled siRNA (Scramble) was used as a control (CTL). Equal amounts of cell lysates were subjected to Western blot. One of three independent experiments is shown.

To further elucidate the regulatory mechanisms controlling the cross-talk between PDGF and TGFβ in the activation of Smad1 and induction of Col4 in MCs, we examined whether LDL receptor related protein-1 (LRP1) is involved in the signal pathways. Because Boucher et al. reported that LRP1 is tightly involved in the pathogenesis of atherosclerosis by regulating signaling of TGFβ and PDGF, and their receptors [27], [28], knockdown analysis using LRP1 siRNAs was perfomed to examine the role of LRP1 in the regulatory effect of PDGF-induced Col4 expression and PDGF-activated TGFβ signaling pathway in MCs. Knockdown of LRP1 enhanced the downstream pathway of PDGF (Figure 7A) with the exception of ALK1 (Figure 7B). These results suggest that LRP1 has a significant inhibitory effect on PDGF signaling pathway leading to production of Col4 in MCs.

### PDGF signaling pathway is partially involved in the AngII-induced c-Src/Smad1 signal activation in MCs

**Figure 8:**
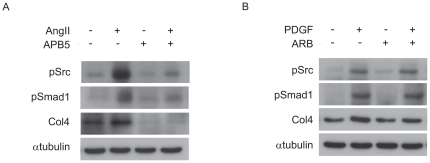
Molecular cross-talk between PDGF and AngII signaling pathways in MCs. (A) Effects of APB5 on pSrc, pSmad1 and Col4. MCs were preincubated with APB5 (100 ng/ml) or control rat IgG for 24 h before exposure to AngII (0.1 µM, 30 min). (B) Effects of olmesartan (ARB) on pSrc, pSmad1 and Col4. MCs were preincubated with olmesartan (10 µM) or methanol for 48 h before exposure to PDGF (5 ng/ml, 12 h). Equal amounts of cell lysates were subjected to Western blot. One of three independent experiments is shown.

We previously reported that AngII activates the c-Src/Smad1 signaling pathway in the development of diabetic nephropathy and cultured MCs [23]. To investigate whether AngII signals influence the regulatory mechanisms of PDGF-induced c-Src/Smad1 signal transduction, we examined the inhibitory effects of APB5 and AngII receptor blocker (ARB) on the activation of c-Src, Smad1, and Col4 by AngII and PDGF, respectively. APB5 clearly attenuated the AngII-induced c-Src/Smad1/Col4 signal (Figure 8A). In contrast, ARB treatment slightly reduced PDGF-induced activation of the signal (Figure 8B). These data suggest that PDGF signaling pathway is activated by AngII in MCs.

## Discussion

Cellular proliferation and extracellular matrix accumulation are characteristic features of progressive glomerular diseases, a major cause of end-stage renal failure in humans throughout much of the world. Glomerulosclerosis followed by mesangial proliferative glomerulonephritis is characterized by mesangial matrix expansion and phenotypic change of MCs [3]. In the expanded mesangial matrix, Col4 is a major component of ECM and is overproduced in glomerulosclerosis [6]. In addition, phenotypic modulation is a commonly observed feature in the progression of many renal diseases leading to CKD and ESRD. Expression of SMA is a well-known marker for the activation of MCs in most glomerular diseases. We previously reported that Smad1 upregulated the expression of Col4 and SMA [5], [6] and thereby participates in the development of glomerulosclerosis in experimental glomerulonephritis [4]. However, the molecule that activates Smad1 in glomerulonephritis has not been fully elucidated. Since PDGF has been consistently implicated in cell proliferation and extracellular matrix accumulation, which characterize progressive glomerular disease [29], and since c-Src is an important component of the PDGF signaling pathway [30], we first investigated whether c-Src is induced in glomeruli of proliferative glomerulonephritis. In Thy1 GN, Col4 is strongly expressed in the sclerotic lesions of glomeruli, as previously described [4], [21]. We show here that c-Src and Smad1 are heavily phosphorylated in the nuclei of glomerular cells in Thy1 GN. This phosphorylation parallels the progress of glomerulosclerosis and peaks on day 6, when Col4 and SMA expression levels have peaked. These results suggest that c-Src has a potential to be involved in the development of glomerulosclerosis in mesangial proliferative glomerulonephritis.

c-Src was identified as the first proto-oncogene, and a great deal of work has been carried out to elucidate its role in biological systems [31]–[33]. The two main areas in which Src inhibitors have been applied are regulating bone resorption [34], [35] and both tumor growth and metastasis [36], [37]. Most previous studies have shown that the role of Src family members is related to inflammatory responses. Additionally, the small chemical inhibitors that effectively and specifically block Src kinases could have great clinical implications for diseases with acute inflammatory responses [38], [39]. In a rat renal ischemia-reperfusion injury model, increased active Src expression was found in the injured rat kidney after reperfusion [40]. To our knowledge, however, no report has demonstrated that c-Src is involved in the development of glomerulosclerosis in glomerular diseases. In the rat proliferative glomerulonephritis model, administration of PP2 completely abolished the phosphorylation of c-Src and Smad1 and resulted in the amelioration of glomerulosclerosis. Therefore, the activation of c-Src signal transduction plays a pivotal role in glomerulosclerosis, implicating it as a novel target of the therapeutic strategies for glomerulonephritis. Moreover, our findings show a new side of PP2 as an anti-glomerular disease agent.

**Figure 9:**
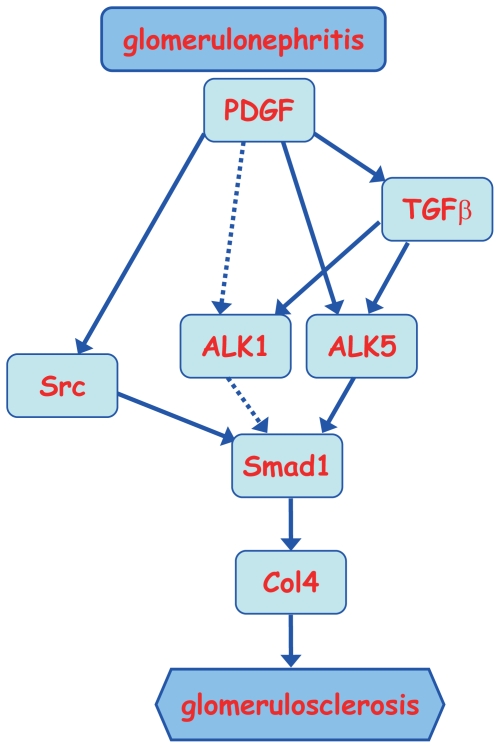
Proposed model for PDGF effects on Smad1 activation and Col4 expression in glomerulonephritis. Activation of Smad1 by PDGF mediates at least two different signal transduction pathways, TGFβ-ALK5-Smad1 and Src-Smad1. ALK1 may potentially activate Smad1 when exposed to high concentration of PDGF (broken arrows). The expression of ALK5 is induced by PDGF and is largely independent of TGFβ. Excessive activation of these signaling pathways may result in Col4 overproduction leading to the development of glomerulosclerosis in glomerulonephritis.

In addition, PDGF is known to contribute to the development of both experimental and human glomerulonephritis [12], [13]. Src kinase activation has been reported to contribute to PDGF-dependent cell-cycle proliferation, mitogenesis, and chemotaxis [24], [29], [30]. Thus, to investigate the molecular mechanisms underlying the progression of proliferative glomerulonephritis, we used cultured MCs under PDGF stimulation. PDGF induced phosphorylation of c-Src and Smad1 as well as Col4 expression, and these changes were blocked by PP2. The interaction between PDGFR and c-Src may be important for the phosphorylation of c-Src. In addition, the siRNA silencing experiments confirmed that c-Src regulated Smad1 activation. These findings suggest that c-Src activation is a key event in the PDGF-induced phosphorylation of Smad1, followed by the subsequent overproduction of Col4 in proliferative glomerulonephritis. In addition, PDGF activated TGFβ signaling pathways by induction of TGFβ and its type I receptors, ALK1 and ALK5. In particular, the induction of ALK1 may be an important event, because ALK1 transduce TGFβ signals to Smad1. Furthermore, several recent reports demonstrated that LRP1 has an inhibitory effect on TGFβ signaling pathway as well as PDGF signaling pathway [27], [28]. As expected, LRP1 silencing exhibited additional effect on the activation of TGFβ signals by PDGF. Hence, LRP1 represents a promising new therapeutic target for the control of proliferative glomerular diseases. Moreover, our previous study demonstrated that AngII stimulated this Src-Smad1 axis independent of p44/42 MAP kinase activation and that the AngII receptor blocker ARB blocked this pathway. Because it is generally accepted that the AngII blockade significantly delays the progression of proliferative glomerulonephritis [41], [42], our previous findings implied that the inhibition of the Src-Smad1 axis may partially explain the AngII-induced progression of proliferative glomerunonephritis. PDGF-induced activation of c-Src/Smad1 signaling pathway leading to Col4 production also plays an important role downstream of AngII stimulation, whereas ARB treatment did not fully suppressed the effect of PDGF. Chemical inhibitors directly or indirectly targeting Src kinases have been developed as potential drugs for the treatment of cancer [43]. It was recently reported that the inhibition of c-Src by these chemical inhibitors helps to prevent ischemia-reperfusion-induced injury in organs [38], [39]. The present study raises the possibility that using these chemical inhibitors to block Src signal transduction could be a promising option for ameliorating proliferative glomerulonephritis as well as for the already reported effects of these inhibitors on excessive inflammatory cells, monocytes and macrophages [44], [45]. Another report by Severgnini et al. demonstrated that c-Src controls STAT3 activation in acute lung injury [46]. In addition, we previously reported that STAT3 is involved in the development of glomerulosclerosis in experimental proliferative glomerulonephritis [4]. In light of these previous findings, our results highlight the importance of c-Src in the development of glomerulosclerosis in glomerulonephritis. Combining with our overall findings summarized in Figure 9, we can speculate that Smad1-mediated production of Col4 leading to mesangial expansion is a critical event in the development of glomerulosclerosis.

In conclusion, our present study indicates that c-Src activates Smad1-induced ECM production and phenotypic alteration, and is involving in the progression of proliferative glomerulonephritis leading to glomerulosclerosis. Further understanding of the Src/Smad1 pathway and the molecules involve in this pathway is critical for the clarification of glomerulosclerosis and to pave the way for a strategy to treat progressive glomerulonephritis.

## Supporting Information

Figure S1 **Time course of renal function in Thy1 GN.** Urine volume (*P = 0.042) (A), serum BUN (*P = 0.014) (B), and UAE (*P = 0.017) (C) in Thy1 GN. Data represent mean values ± S.D. of at least three independent experiments; n = 6 for each experimental group.
(TIF)

Figure S2 **Knockdown of c-Src expression.** MCs were transfected with three different siRNAs specific for c-Src and with scrambled siRNA with or without PDGF stimulation. Effects of RNAi-mediated silencing of c-Src on pSrc, pSmad1 and Col4 under stimulation of PDGF (5 ng/ml, 12 h) were analyzed by Western blot. GAPDH served as a loading control.
(TIF)

## References

[1] Fogo A, Ichikawa I (1989). Evidence for a central role of glomerular growth in the development of sclerosis.. Semin Nephrol.

[2] Striker LJ, Doi T, Elliot S, Striker GE (1989). The contribution of glomerular mesangial cells to progressive glomerulosclerosis.. Semin Nephrol.

[3] Kagami S, Border WA, Miller DE, Noble NA (1994). Angiotensin II stimulates extracellular matrix protein synthesis through induction of transforming growth factor-beta expression in rat glomerular mesangial cells.. J Clin Invest.

[4] Takahashi T, Abe H, Arai H, Matsubara T, Nagai K (2005). Activation of STAT3/Smad1 is a key signaling pathway for progression to glomerulosclerosis in experimental glomerulonephritis.. J Biol Chem.

[5] Abe H, Matsubara T, Iehara N, Nagai K, Takahashi T (2004). Type IV collagen is transcriptionally regulated by Smad1 under advanced glycation end product (AGE) stimulation.. J Biol Chem.

[6] Matsubara T, Abe H, Arai H, Nagai K, Mima A (2006). Expression of Smad1 is directly associated with mesangial matrix expansion in rat diabetic nephropathy.. Lab Invest.

[7] Doi T, Vlassara H, Kirstein M, Yamada Y, Striker GE (1992). Receptor-specific increase in extracellular matrix production in mouse mesangial cells by advanced glycosylation end products is mediated via platelet-derived growth factor.. Proc Natl Acad Sci USA.

[8] Tallquist M, Kazlauskas A (2004). PDGF signaling in cells and mice.. Cytokine Growth Factor Rev.

[9] Leveen P, Pekny M, Gebre-Medhin S, Swolin B, Larsson E (1994). Mice deficient for PDGF B show renal, cardiovascular, and hematological abnormalities.. Genes Dev.

[10] Lindahl P, Hellstrom M, Kalen M, Karlsson L, Pekny M (1998). Paracrine PDGF-B/PDGF-Rbeta signaling controls mesangial cell development in kidney glomeruli.. Development.

[11] Soriano P (1994). Abnormal kidney development and hematological disorders in PDGF beta-receptor mutant mice.. Genes Dev.

[12] Floege J, Eitner F, Alpers CE (2008). A new look at platelet-derived growth factor in renal disease.. J Am Soc Nephrol.

[13] Johnson RJ, Floege J, Couser WG, Alpers CE (1993). Role of platelet-derived growth factor in glomerular disease.. J Am Soc Nephrol.

[14] Johnson RJ, Raines EW, Floege J, Yoshimura A, Pritzl P (1992). Inhibition of mesangial cell proliferation and matrix expansion in glomerulonephritis in the rat by antibody to platelet-derived growth factor.. J Exp Med.

[15] Brown MT, Cooper JA (1996). Regulation, substrates and functions of src.. Biochim Biophys Acta.

[16] Schlessinger J (2000). New roles for Src kinases in control of cell survival and angiogenesis.. Cell.

[17] Waters CM, Connell MC, Pyne S, Pyne NJ (2005). c-Src is involved in regulating signal transmission from PDGFbeta receptor-GPCR(s) complexes in mammalian cells.. Cell Signal.

[18] Silver BJ, Jaffer FE, Abboud HE (1989). Platelet-derived growth factor synthesis in mesangial cells: induction by multiple peptide mitogens.. Proc Natl Acad Sci U S A.

[19] Floege J, Eng E, Young BA, Alpers CE, Barrett TB (1993). Infusion of platelet-derived growth factor or basic fibroblast growth factor induces selective glomerular mesangial cell proliferation and matrix accumulation in rats.. J Clin Invest.

[20] MacKay K, Striker LJ, Elliot S, Pinkert CA, Brinster RL (1988). Glomerular epithelial, mesangial, and endothelial cell lines from transgenic mice.. Kidney Int.

[21] Makibayashi K, Tatematsu M, Hirata M, Fukushima N, Kusano K (2001). A vitamin D analog ameliorates glomerular injury on rat glomerulonephritis.. Am J Pathol.

[22] Mima A, Arai H, Matsubara T, Abe H, Nagai K (2008). Urinary Smad1 is a novel marker to predict later onset of mesangial matrix expansion in diabetic nephropathy.. Diabetes.

[23] Mima A, Matsubara T, Arai H, Abe H, Nagai K (2006). Angiotensin II-dependent Src and Smad1 signaling pathway is crucial for the development of diabetic nephropathy.. Lab Invest.

[24] Yamamoto Y, Kato I, Doi T, Yonekura H, Ohashi S (2001). Development and prevention of advanced diabetic nephropathy in RAGE-overexpressing mice.. J Clin Invest.

[25] Throckmorton DC, Brogden AP, Min B, Rasmussen H, Kashgarian M (1995). PDGF and TGF-beta mediate collagen production by mesangial cells exposed to advanced glycosylation end products.. Kidney Int.

[26] Pan D, Yang J, Lu F, Xu D, Zhou L (2007). Platelet-derived growth factor BB modulates PCNA protein synthesis partially through the transforming growth factor beta signalling pathway in vascular smooth muscle cells.. Biochem Cell Biol.

[27] Boucher P, Gotthardt M, Li WP, Anderson RG, Herz J (2003). LRP: role in vascular wall integrity and protection from atherosclerosis. Science.

[28] Boucher P, Li WP, Matz RL, Takayama Y, Auwerx J (2007). LRP1 functions as an atheroprotective integrator of TGFbeta and PDFG signals in the vascular wall: implications for Marfan syndrome.. PLoS One.

[29] Floege J, Johnson RJ (1995). Multiple roles for platelet-derived growth factor in renal disease.. Miner Electrolyte Metab.

[30] Kypta RM, Goldberg Y, Ulug ET, Courtneidge SA (1990). Association between the PDGF receptor and members of the src family of tyrosine kinases.. Cell.

[31] Stehelin D, Varmus HE, Bishop JM, Vogt PK (1976). DNA related to the transforming gene(s) of avian sarcoma viruses is present in normal avian DNA.. Nature.

[32] Golden A, Nemeth SP, Brugge JS (1986). Blood platelets express high levels of the pp60c-src-specific tyrosine kinase activity.. Proc Natl Acad Sci USA.

[33] Soriano P, Montgomery C, Geske R, Bradley A (1991). Targeted disruption of the c-src proto-oncogene leads to osteopetrosis in mice.. Cell.

[34] Lowe C, Yoneda T, Boyce BF, Chen H, Mundy GR (1993). Osteopetrosis in Src-deficient mice is due to an autonomous defect of osteoclasts.. Proc Natl Acad Sci USA.

[35] Tanaka S, Amling M, Neff L, Peyman A, Uhlmann E (1996). c-Cbl is downstream of c-Src in a signalling pathway necessary for bone resorption.. Nature.

[36] Trevino JG, Summy JM, Lesslie DP, Parikh NU, Hong DS (2006). Inhibition of SRC expression and activity inhibits tumor progression and metastasis of human pancreatic adenocarcinoma cells in an orthotopic nude mouse model.. Am J Pathol.

[37] Mandal M, Myers JN, Lippman SM, Johnson FM, Williams MD (2008). Epithelial to mesenchymal transition in head and neck squamous carcinoma: association of Src activation with E-cadherin down-regulation, vimentin expression, and aggressive tumor features.. Cancer.

[38] Severgnini M, Takahashi S, Tu P, Perides G, Homer RJ (2005). Inhibition of the Src and Jak kinases protects against lipopolysaccharide-induced acute lung injury.. Am J Respir Crit Care Med.

[39] Okutani D, Lodyga M, Han B, Liu M (2006). Src protein tyrosine kinase family and acute inflammatory responses.. Am J Physiol Lung Cell Mol Physiol.

[40] Takikita-Suzuki M, Haneda M, Sasahara M, Owada MK, Nakagawa T (2003). Activation of Src kinase in platelet-derived growth factor-B-dependent tubular regeneration after acute ischemic renal injury.. Am J Pathol.

[41] Peters H, Border WA, Noble NA (1998). Targeting TGF-beta overexpression in renal disease: maximizing the antifibrotic action of angiotensin II blockade.. Kidney Int.

[42] Catapano F, Chiodini P, De Nicola L, Minutolo R, Zamboli P (2008). Antiproteinuric response to dual blockade of the renin-angiotensin system in primary glomerulonephritis: meta-analysis and metaregression.. Am J Kidney Dis.

[43] Spreafico A, Schenone S, Serchi T, Orlandini M, Angelucci A (2008). Antiproliferative and proapoptotic activities of new pyrazolo[3,4-d]pyrimidine derivative Src kinase inhibitors in human osteosarcoma cells.. FASEB J.

[44] Huang WC, Chen JJ, Chen CC (2003). c-Src-dependent tyrosine phosphorylation of IKKbeta is involved in tumor necrosis factor-alpha-induced intercellular adhesion molecule-1 expression.. J Biol Chem.

[45] Huang WC, Chen JJ, Inoue H, Chen CC (2003). Tyrosine phosphorylation of I-kappa B kinase alpha/beta by protein kinase C-dependent c-Src activation is involved in TNF-alpha-induced cyclooxygenase-2 expression.. J Immunol.

[46] Severgnini M, Takahashi S, Rozo LM, Homer RJ, Kuhn C (2004). Activation of the STAT pathway in acute lung injury.. Am J Physiol Lung Cell Mol Physiol.

