# Correction: Activation of Src Mediates PDGF-Induced Smad1 Phosphorylation and Contributes to the Progression of Glomerulosclerosis in Glomerulonephritis

**DOI:** 10.1371/annotation/b387ee8b-c672-4c19-9871-e5a4f6a59f57

**Published:** 2012-09-11

**Authors:** Akira Mima, Hideharu Abe, Kojiro Nagai, Hidenori Arai, Takeshi Matsubara, Makoto Araki, Kazuo Torikoshi, Tatsuya Tominaga, Noriyuki Iehara, Atsushi Fukatsu, Toru Kita, Toshio Doi

There were errors in Figures 2, 4, and 7. 

-The correct Figure 2 can be seen here: 

**Figure:**
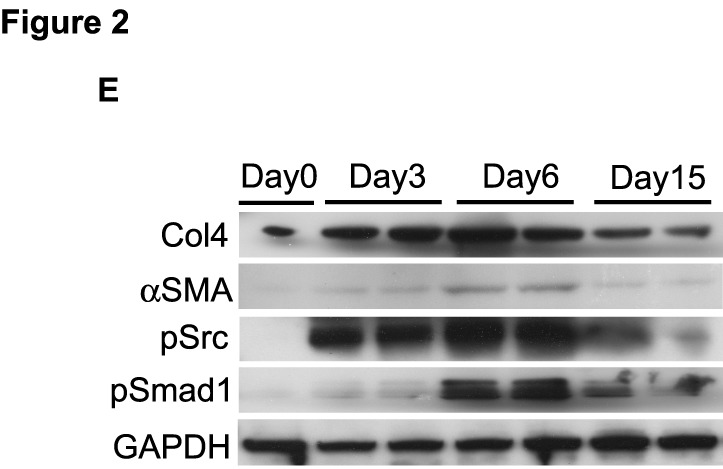


[^]

-In Figure 4 B-D, the y-axis was incorrectly labeled “GAPDH.” The correct labels are “* tubulin.”

-The correct Figure 7 can be seen here: 

**Figure:**
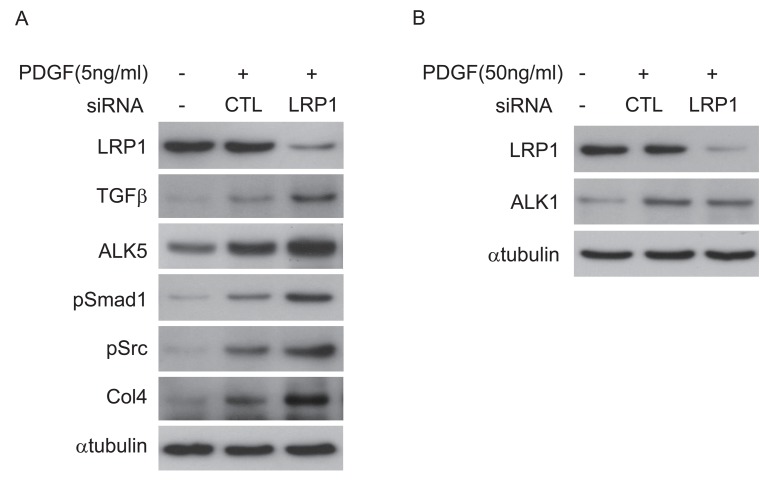


[^] 

