# Supplementary figures and images for: Activation of Src Mediates PDGF-Induced Smad1 Phosphorylation and Contributes to the Progression of Glomerulosclerosis in Glomerulonephritis
Source: PLoS One. 2011 Mar 22;6(3):e17929. doi: 10.1371/journal.pone.0017929 (PMC3062564; doi:10.1371/journal.pone.0017929)

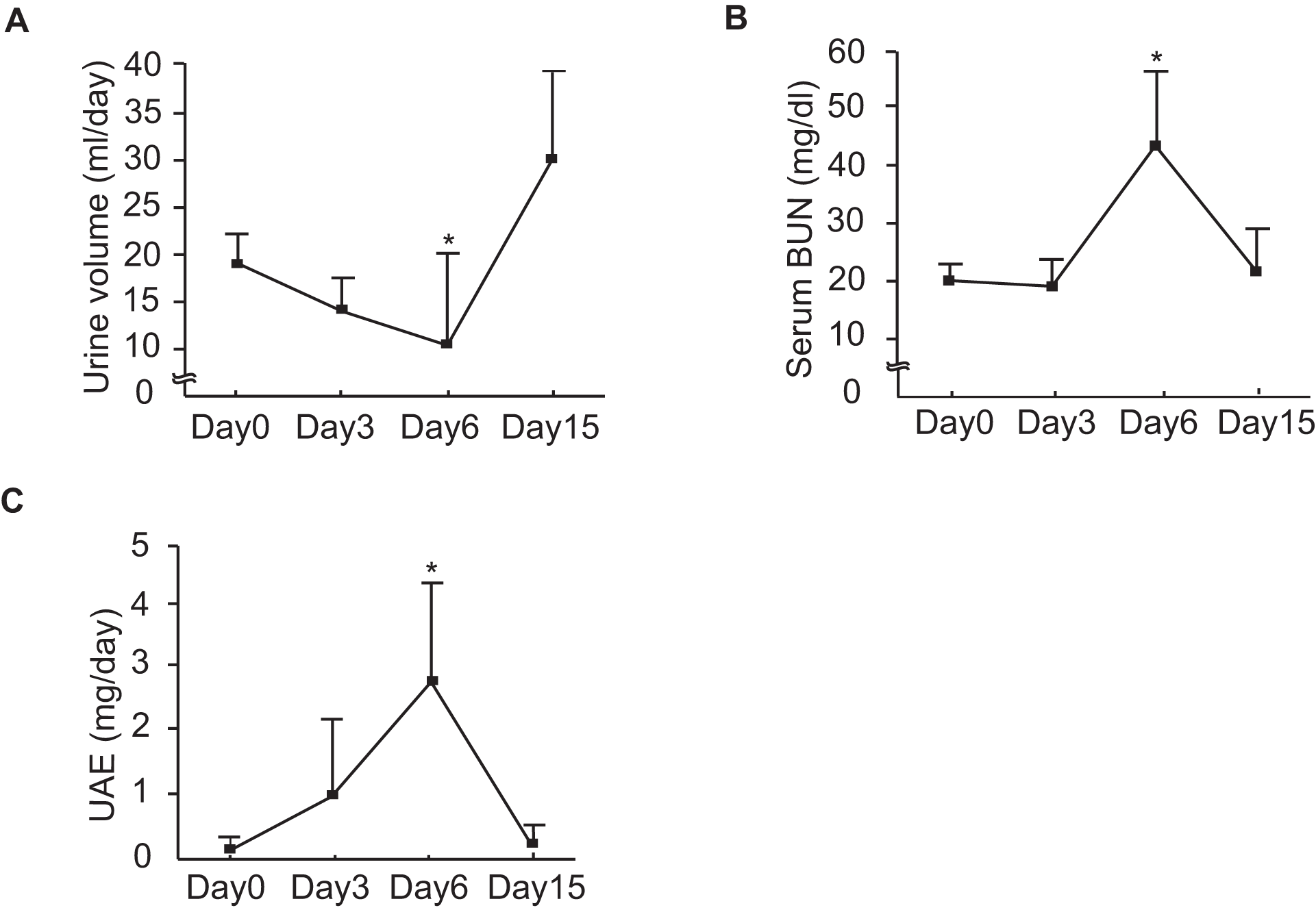

Supplement: Figure S1 — Time course of renal function in Thy1 GN. Urine volume (*P = 0.042) (A), serum BUN (*P = 0.014) (B), and UAE (*P = 0.017) (C) in Thy1 GN. Data represent mean values ± S.D. of at least three independent experiments; n = 6 for each experimental group. (TIF) [file pone.0017929.s001.tif]

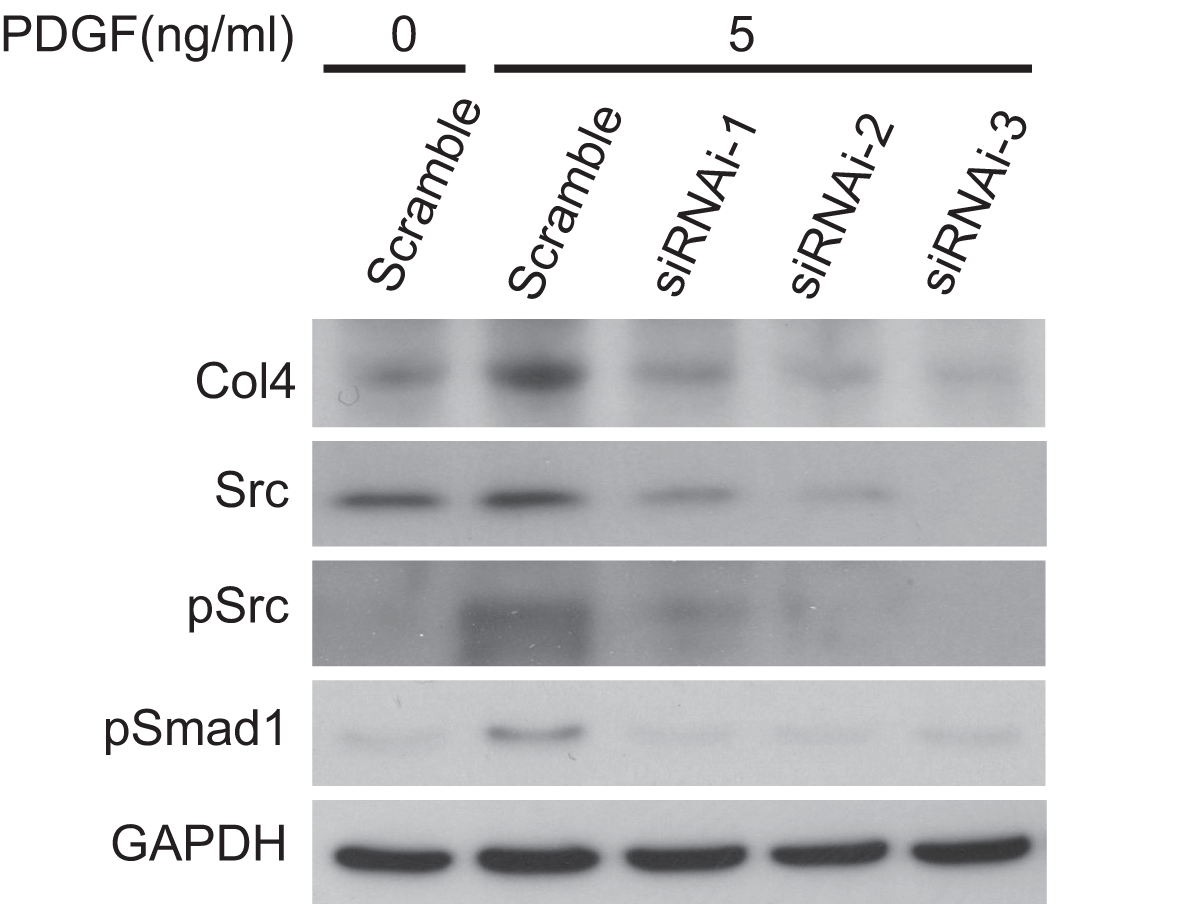

Supplement: Figure S2 — Knockdown of c-Src expression. MCs were transfected with three different siRNAs specific for c-Src and with scrambled siRNA with or without PDGF stimulation. Effects of RNAi-mediated silencing of c-Src on pSrc, pSmad1 and Col4 under stimulation of PDGF (5 ng/ml, 12 h) were analyzed by Western blot. GAPDH served as a loading control. (TIF) [file pone.0017929.s002.tif]
